# Sustainability in surgery: insights from the 2024 SAGES Annual Meeting

**DOI:** 10.1007/s00464-026-13181-x

**Published:** 2026-08-10

**Authors:** Anjelli Wignakumar, Justin Dourado, Sameh Hany Emile, Julio F. Fiore, Patricia Sylla, Steven D. Wexner, Lakshmi Linkeshwaran, Marylise Boutros, Olivia Monton

**Affiliations:** 1 Department of Colorectal Surgery, Ellen Leifer Shulman and Steven Shulman Digestive Disease Center, Cleveland Clinic Florida, Weston, FL USA; 2 Department of General Surgery, Baptist Heath South Florida, Florida Atlantic University, Boca Raton, FL USA; 3 Colorectal Surgery Unit, General Surgery Department, Mansoura University Hospitals, Mansoura, Egypt; 4 Department of Surgery, McGill University, Montreal, QC Canada; 5 Division of Colon and Rectal Surgery, Mount Sinai Health System, New York, NY USA; 6 Department of Colorectal Surgery, Medstar Georgetown University Health, Washington, DC USA; 7 University College London, London, UK; 8 Department of Surgery, McMaster University, Hamilton, ON Canada; 9 Department of Epidemiology, Johns Hopkins Bloomberg School of Public Health, Baltimore, MD USA; 10 Cleveland Clinic Florida, Weston Hospital, 2950 Cleveland Clinic Boulevard, Weston, FL 33331 USA

**Keywords:** Sustainability, Surgery, SAGES Annual Meeting, Interviews

## Abstract

**Background:**

The healthcare sector is a major contributor to global carbon emissions, with operating rooms representing particularly waste-intensive environments. Despite growing interest in sustainability in surgery, little qualitative research has explored surgeons’ and surgical trainees’ perceptions of this issue or their responses to professional society sustainability initiatives.

**Methods:**

This qualitative study used semi-structured, in-depth interviews with surgeons and surgical trainees who attended at least one sustainability session at the 2024 Society of American Gastrointestinal and Endoscopic Surgeons (SAGES) Annual Meeting. Purposive sampling was used to recruit participants, and interviews were conducted in person or via Zoom. Transcripts were analyzed using the framework method with an abductive approach to coding. Domains and themes were generated using constant comparison.

**Results:**

18 participants were interviewed, including surgeons, fellows, and residents from academic and community settings. Two major domains emerged: (1) attitudes and perceptions of sustainability in surgery and (2) responses to the 2024 SAGES Annual Meeting’s sustainability agenda. Participants conceptualized sustainability as both mindful resource use and concrete waste-reduction actions. The operating room was widely viewed as a highly wasteful environment. Sustainability sessions were perceived as educational, motivating, and impactful, particularly through leadership engagement and the presentation of quantitative environmental data. Many participants reported increased awareness and expressed intentions to implement sustainable changes in their own practices.

**Conclusions:**

Participants demonstrated strong support for sustainability initiatives and viewed the SAGES sustainability sessions as a catalyst for individual and institutional change. These findings highlight the influential role of professional societies, leadership, and data-driven education in advancing sustainability in surgical practice.

**Supplementary Information:**

The online version contains supplementary material available at https://doi.org/10.1007/s00464-026-13181-x.

The relationship between healthcare and climate change has been described as mutual, whereby “climate change threatens public health, and healthcare contributes to climate change” [1]. Healthcare significantly contributes to environmental degradation through waste production, water and electricity consumption, gas emissions, and transportation [2,3]. It accounts for approximately 4.4% of net carbon emissions globally [4,5] and 8.5% of total greenhouse gas emissions in the United States [6]. If current practices continue, the healthcare sector’s carbon footprint is projected to triple by 2050 [7], a trajectory that some argue contradicts the medical profession’s pledge to “first, do no harm.” Global warming is already fueling a cascade of adverse health effects, from worsening air quality to food and water insecurity and the spread of infectious disease. In this context, the healthcare sector’s own emissions contribute directly to both human harm and the erosion of environmental sustainability.

Operating rooms (ORs) are a significant contributor to climate impact, generating more than 50% of all hospital waste in the United States and worldwide [8,9]. The substantial greenhouse effect of anesthetic gases further exacerbates the environmental footprint of surgical care [10]. There have been numerous initiatives to improve sustainability within ORs [10,11]. Sullivan et al. recommended that institutions begin with low-investment initiatives, such as creating ‘lean packs’ of surgical instruments in an attempt to reduce waste. Wormer et al. reported that the implementation of energy-saving behaviors, such as powering down OR equipment at night, could not only reduce CO2 emissions by 234.3 metric tons per year but also save institutions up to $33,000 annually [9]. A recent scoping review found that educational initiatives, recycling programs, and efforts to limit the use of anesthetic gases have the greatest impact in reducing the carbon footprint of surgical care [10]. Surgical societies have also begun to take a more active role. In 2023, the Society of American Gastrointestinal and Endoscopic Surgeons (SAGES) and the European Association for Endoscopic Surgery (EAES) formed a joint task force on Sustainability in Surgical Practice, which seeks to advance sustainability in surgery through education, mitigation strategies, and innovation [12].

Furthermore, SAGES placed a strong emphasis on sustainability at its 2024 Annual Meeting. The theme of the meeting was “Innovate and Collaborate for a Sustainable Future,” reflecting the society’s mission to promote sustainability within surgery. The meeting featured a dedicated half-day program on sustainability in surgery, which included the Presidential Address, a plenary session, a multidisciplinary panel, and a scientific session. Furthermore, conference attendees were encouraged to share their perspectives on social media using the “#sustainableSAGES” hashtag. Through these efforts, SAGES leadership aimed to educate members on the environmental impact of surgical practices and inspire change from conference attendees.

There is increasing research investigating surgeons’ attitudes toward sustainability in surgery. A 2024 international survey study was conducted by the SAGES-EAES Sustainability in Surgical Practice task force to explore surgeons’ attitudes toward mitigating the impact of surgical practice on environmental sustainability. Only 10% of the 1024 respondents from North America and Europe could accurately estimate the carbon footprint of surgical activities; however, more than 60% of respondents expressed motivation to practice sustainability [13].

Despite increasing evidence in this field, there is limited qualitative research exploring surgeons’ and surgical trainees’ perceptions of environmental sustainability within surgery. Additionally, responses to the 2024 SAGES Annual Meeting’s sustainability agenda remain unexplored. This study aimed to explore their perceptions of (1) sustainability in surgery and (2) the SAGES Annual Meeting’s sustainability agenda, including reactions to the sustainability sessions and perceptions of the society’s broader role in advancing sustainability in surgical care.

## Methods

This qualitative study was reported in accordance with the Consolidated Criteria for Reporting Qualitative Research (COREQ) (Supplementary Material, Table A1) [14]. The study was deemed exempt by the Mount Sinai Hospital Institutional Review Board (STUDY-24-01222).

### Participants

In-depth individual interviews were conducted at and following the 2024 SAGES Annual Meeting in Cleveland, OH in April 2024. Purposive sampling was used to identify staff surgeons, surgical fellows, and surgical residents who attended at least one of the sustainability sessions at the SAGES Annual Meeting. Attendees who were not staff surgeons or surgical trainees, including industry representatives, and those who did not attend a sustainability session were excluded. A minimum sample size of 12 participants was targeted, guided by Guest et al.’s recommendation [15] for sample sizes in qualitative interview studies using non-probabilistic sampling.

### Recruitment

Eligible participants were invited to participate in the study via a QR code that was displayed on posters at the registration desk and around the conference venue, as well as on a slide displayed on the main screen during the Karl Storz Lecture and the Presidential Address. The QR code was linked to a SurveyMonkey (San Mateo, California) form, which described the study in more detail and included information about scheduling individual interviews, as well as a demographic survey for those who scheduled interviews.

### Interview guide

The interview guide was developed by five members of the research team, including one female clinical research fellow (A.W.), one male surgical resident (J.D.), two female colorectal surgeons (P.S., M.B.), and one female surgical resident with formal training in qualitative research (O.M.). The interview guide spanned several key content areas, including (1) perceptions of sustainability in surgery, (2) approaches to incorporating sustainability into surgical practice, (3) barriers and facilitators to sustainability in surgery, and (4) perceptions of the sustainability sessions at the SAGES Annual Meeting.

### Interview procedures

Two authors (A.W., J.D.) conducted the semi-structured interviews after completing training in qualitative interviewing. Both held positions within the colorectal surgery department at Cleveland Clinic Florida at the time of the interviews. Most interviews were conducted in person at the SAGES Annual Meeting, while a few interviews were conducted using a secure version of Zoom (Zoom Video Communications, Inc., San Jose, CA) after the conference. The Zoom-based format was utilized for eligible participants who signed up but could not physically attend any of the available interview slots at the SAGES Annual Meeting. To minimize recall bias, all Zoom interviews were conducted within 7 days of the conference. Oral informed consent was obtained from all participants prior to the interviews. Each interview lasted approximately 20–30 min and was conducted in English. At the end of each interview, participants were afforded the opportunity to ask questions or clarify any of their responses. All interviews were audio-recorded and transcribed verbatim. Interview transcripts were stored on password-protected computers at Cleveland Clinic Florida. Authors who held leadership positions at SAGES at the time of the interviews (S.W., P.S., M.B.) were not involved in conducting interviews.

### Data analysis

Interview transcripts were imported into MAXQDA Version 2024 (VERBI Software, Berlin, Germany). The analysis was performed using the framework method described by Gale et al [16] and included familiarization with the data, identifying codes and coding the data, generating domains and themes, and drawing inferences. An abductive approach to coding was adopted, with codes developed deductively and inductively based on concepts included in the interview guide and the data [17]. The codebook was tested by two authors (A.W., J.D.), who each coded two transcripts. It was then reviewed and finalized by three authors (A.W., J.D., O.M.), who subsequently coded all the transcripts independently. The coders met at regular intervals to ensure consistency and resolve discrepancies in the coding approach. Two authors (A.W., O.M.) then created an analysis table using codes related to individual attitudes and perceptions of sustainability in surgery and the SAGES Annual Meeting sustainability sessions. The data were then summarized into the following categories: (1) understanding, interest, and engagement in sustainability, (2) motivation for attending the sustainability sessions, (3) general impressions of the sustainability sessions, and (4) lessons learned from the sustainability sessions. The same two authors (A.W., O.M.) then applied constant comparison to generate domains and associated themes. Comparisons by informant type (surgeon, fellow, resident) were not performed. Authors holding leadership positions at SAGES at the time of the analysis (S.W., P.S., M.B.) did not serve as data analysts.

## Results

Our sample included 18 participants: 6 general surgeons, 4 colorectal surgeons, 5 general surgery residents, and 3 fellows. One fellow was specializing in colorectal surgery, while the other 2 were completing minimally invasive surgery fellowships. Twelve participants (67%) worked at academic centers, 4 (22%) at community hospitals, 1 in private practice, and 1 at a Veterans Affairs hospital. Seven participants were aged between 25 and 34 years, and 6 were 55 years or older. A summary of the participants’ characteristics is provided in Table 1.

**Table 1:** Participant characteristics

Characteristic	*n* (%)
Type of informant	Of 18:
Resident	5 (28%)
Fellow	3 (17%)
Surgeon	10 (56%)
Specialty	Of 10:
General surgery	6 (60%)
Colorectal surgery	4 (40%)
Setting	Of 18:
Academic center	12 (67%)
Community hospital	4 (22%)
Other	2 (11%)
Age	Of 18:
25–34	7 (39%)
35–44	2 (11%)
45–54	3 (17%)
55–64	4 (22%)
65 +	2 (11%)

We identified two overarching domains, each with several associated themes: (1) attitudes and perceptions of sustainability in surgery and (2) responses to the 2024 SAGES Annual Meeting’s sustainability agenda.

### Attitudes and perceptions of sustainability in surgery

#### Conceptualizing sustainability in surgery

Participants conceptualized sustainability in different ways, ranging from reducing waste and conserving energy to adopting a broader mindset of environmental responsibility within surgery. When asked how they would describe sustainability in surgery, most participants discussed concrete actions that would reduce the carbon footprint of surgical practice, such as reducing waste, decreasing greenhouse gas emissions, conserving water, and limiting the use of plastic. For example, one resident described sustainability as “Trying to make surgery and the use of surgical resources more efficient and looking to reduce waste and excess where possible in the preoperative, operative, and postoperative phases of care.” (**KI15_Resident**) The perception of sustainability in surgery as reducing waste within the surgical setting, particularly the OR, was highlighted by many participants.

Several participants framed sustainability as mindfulness in resource use, emphasizing the importance of being both intentional and conscious in how surgical resources were utilized. One surgeon described sustainability in surgery as being “more conscious of our resource utilization in surgery, so that we don’t expend more resources than we need to do a good job.” (**KI06_Surgeon**) Similarly, another surgeon described it as “Being able to preserve what we do in practice and treat our patients without harming or impacting the environment.” (**KI09_Surgeon**) These two informants emphasized the balance between resource use and maintaining surgical proficiency.

Few informants also highlighted the potential role of resource availability in surgical waste production. One surgeon, who had previously worked in a low-resource setting and was currently practicing in the United States, described differences in resource availability between the two settings. They thought that resource availability in the United States contributed to waste in the OR and reflected: “In [low-income setting], when you are closing a belly, you will think twice before asking for another stitch because they don’t have that many. They don’t have it.” (**KI01_Surgeon**) This informant also shared an example of changing gloves multiple times before the start of a case and explained that the abundance of resources in the United States encouraged more liberal resource use, which in turn led to more waste. A fellow who had also practiced in a low-resource setting shared a similar perspective and framed sustainability as the correct use of “limited” and “costly” resources.

#### Navigating sustainability in the OR

Participants consistently described the OR as a waste-intensive environment, frequently referencing the unnecessary opening of supplies, overuse of disposable items, and excessive packaging. One resident stated: “We open so much [equipment] that we don’t use. You wouldn’t do that at home.” (**KI11_Resident**) Similarly, another resident shared: “There are certain cases that I’m like, ‘Oh why are we opening all these things? Do we really need to use that?’” (**KI15_Resident**) A few participants also expressed their discomfort with the waste generated from robotic surgery. One informant shared that, at their institution, 4 sets of gowns and gloves are consistently opened for every person scrubbing into a robotic case, regardless of the actual need.

Although many participants acknowledged the challenges of practicing sustainability within the OR, many described their individual efforts to reduce waste, including reviewing preference cards and removing instruments that are rarely used, limiting the use of disposable instruments, finding substitutes for disposable instruments, collecting non-sterile supplies from the OR before they are thrown away for use in clinic (e.g., lubricating jelly), and limiting the excessive use of supplies when doing procedures on the ward. Finally, one surgeon described a shift in the way they viewed new products, stating that they had become more selective and conscientious when evaluating new instruments or supplies:

I find myself asking more questions when there are new products—like, ‘Is this single-use, can it be reprocessed, is it recyclable?’ I don’t think I ever asked those questions before. I was like, ‘Okay, new product, I’ll use it!’ But now, when we have to evaluate products because we might switch to them as a system, those are the top questions: ‘Is this biodegradable? How is this going to be recycled? Is it single use?’ (**KI09_Surgeon**)

### Responses to the 2024 SAGES Annual Meeting’s sustainability agenda

#### Motivations for attending the sustainability sessions

Motivations for attending the SAGES sustainability sessions were multifaceted. Many participants were drawn by the perceived importance of the topic. When asked what motivated them to attend the sustainability sessions, one surgeon explained: “The importance of the topic really. It was nice to see [the organization’s] initiative.” (**KI01_Surgeon**) Others were personally interested and saw the sessions as an opportunity to learn more about a topic that remains under-represented at surgical conferences. One resident noted:

Personally, for me it’s just an interesting topic and something that is very worthwhile and also it’s just not a topic that I’ve seen in conferences a huge amount, especially not something like that kind of level. (**KI13_Resident**)

Many participants echoed this sentiment and were happy to see a surgical society prioritize the issue. In addition, some informants expressed a desire to become more knowledgeable on the subject. One trainee’s interest was tied to their perception of sustainability as an emerging priority within surgical education:

You know, as a resident, it’s important to be on the up and up on the most recent developments in surgical education and the movement on what’s moving forward. And obviously, the theme of this year’s program was Sustainable SAGES, so I wanted to be in the know of what things were being done to help promote sustainability in surgical training and in the field of surgery. (**KI14_Resident**)

This resident highlighted that sustainability was becoming an integral part of modern surgical discourse and expressed a desire to be informed. Beyond gaining knowledge, a few informants were interested in learning about practical approaches to sustainability that they could take back to their own practice settings. For example, one surgeon stated:

In my professional life, I’ve thought on many occasions that we don’t do a good job considering sustainability. So, I was interested to see if there were things I could take home from the lectures and maybe make changes. (**KI06_Surgeon**)

#### Educational value and impact of the sustainability sessions

The sustainability sessions delivered information on the environmental impacts of surgical practice and introduced powerful examples of leadership and advocacy. A central highlight cited by many participants was the Presidential Address, which was described as enlightening and motivating. One surgeon stated:

I think that [SAGES president] made a great contribution to the entire surgical society. I think that SAGES is one of the biggest congresses worldwide, so the initiative to talk about this problem in a congress and presidential lecture was an incredible idea to underline that it’s a problem, and we have to make a big effort to find a solution together. (**KI03_Surgeon**)

This informant commended the organization for choosing to highlight environmental issues as a central theme at such a large meeting. Another participant commented on the content included in the Presidential Address, stating:

[SAGES President] really highlighted the important facts. That was really a moment that she was giving a perfect summary of what’s going on, and what we have to do to change. For me, I wish that everyone was attending this lecture. (**KI18_Fellow**)

This participant felt the information was presented clearly and effectively, in an action-oriented manner. Along the same lines, many informants appreciated the presentation of quantitative data in the sustainability sessions, which helped contextualize the environmental impact of surgical practice. One surgeon made a comparison to clinical practice:

That’s what I tell my patients that have ileostomy output. You have to measure [the stoma output], because you think you are doing ok, but you’re not. So, when you get to see that in numbers, it’s confirmation. I think it was important to actually see data and numbers. (**KI01_Surgeon**)

This respondent emphasized how seeing the numbers made the issue more tangible, drawing a parallel to how he encourages patients to track their stoma outputs in the clinical setting to understand their losses. One fellow had a similar reaction to seeing the data, emphasizing that it helped them appreciate the breadth of the environmental damage associated with surgery:

I think it’s astonishing to learn and understand the numbers. I think this would be the most important thing. It’s different to just think about it, and then to actually realize and see the numbers. The costs, the damage, and the long-term consequences of what we do. (**KI17_Fellow**)

Most respondents described having important realizations about the environmental harm associated with surgery and were surprised to learn about the extensive carbon footprint of the healthcare industry, particularly ORs: “First of all, I didn’t know that surgery was so wasteful. I thought it was less wasteful. Quite impressive how wasteful it is.” (**KI16_Fellow**) Others learned for the first time about the environmental impact of anesthetic gases: “I did not know the effect of anesthetic [gases] on the environment, so that was the closest thing to a eureka movement that I had.” (**KI06_Surgeon**) Finally, several informants were also surprised to learn about the environmental impact and cost of sterile water for endoscopic procedures.

#### A catalyst for action

Overall, participants felt that the series raised awareness and acted as a catalyst for action. As one resident put it: “It was a call to action for people who can make a difference within their own health system because I think it’s really hard to impact change from the top.” (**KI15_Resident**) This informant highlighted the importance of instilling change at the individual level, as they perceived it as being more feasible than approaching the issue from a higher level, for example, a health system level. Many other informants described feeling motivated to advocate for and implement sustainable change within their own surgical practices, at the individual level. For example, one surgeon said: "In the OR, I can save a lot, and I can save myself—I mean, through my actions.” (**KI01_Surgeon**) Others provided more specific examples of changes they intended to make upon their return from the meeting, such as decreasing the excessive use of blankets in the OR, and being more conscious of the supplies used on the ward and in the emergency room: “As a resident, I do procedures both on the floor and in the emergency room, and I have to think about what supplies that I need to do for that procedure.” (**KI14_Resident**)

Few surgeons also highlighted a responsibility to not only make changes themselves, but to share what they learned with friends and colleagues in surgery: "I must, at every point, try to transport this message to more and more of my friends, colleagues, and those working under or around me." (**KI10_Surgeon**) Another surgeon also highlighted the potential for coaching around sustainability to promote change: “I can watch and kind of guide others, you know, help them be more mindful. So, you can actually bring the coach into the OR.” (**KI01_Surgeon**)

## Discussion

This qualitative study explored the perceptions of surgeons, fellows, and residents on sustainability in surgery and the 2024 SAGES Annual Meeting’s sustainability agenda. Overall, participants expressed a unified recognition of the importance of sustainability in surgery. Participants described sustainability in surgery as both concrete actions and a mindset of being conscious of resource use within surgery. Many described the OR as a waste-intensive environment and expressed frustration over the unnecessary opening of supplies in the OR. They also described individual efforts to practice surgery more sustainably and provided specific examples of their efforts aimed at reducing waste. All informants responded positively to the SAGES Annual Meeting’s focus on sustainability. There were varying motivations for attending the lectures, including perceived importance, personal interest, and a desire for information and tangible, sustainable solutions. Participants were inspired by the sustainability sessions and highlighted the educational value and impact of the sessions. Many emphasized the importance of quantitative data, while others made important realizations related to the content presented. Finally, for many, the sustainability sessions served as a catalyst for action, whereby informants described their intentions to implement sustainable change at the individual level upon their return to clinical practice.

This study builds on existing literature by providing a novel perspective on sustainability in surgery. Unlike previous survey-based research on surgeons’ attitudes toward sustainability [13, 18,19], this qualitative approach provides a deeper understanding of how surgeons and surgical trainees perceive, define, and engage with sustainability in their professional environments. While previous survey studies have shown that many surgeons are motivated to adopt sustainable practices, they often feel uninformed about how to implement such change [13]. This study expands on this by exploring how surgeons conceptualize and navigate sustainability in practice. Furthermore, the use of semi-structured interviews provided valuable feedback on the content and delivery of the sustainability sessions at the SAGES Annual Meeting and captured informants’ views on the role of professional societies in promoting environmental stewardship.

Building on the proactive steps taken by SAGES in championing sustainability within the surgical community, this study offers insights that may support ongoing efforts to integrate environmentally responsible practices into surgical care. SAGES has exemplified leadership by raising awareness of environmental issues and facilitating important discussions within the surgical community. The insights gained from this qualitative analysis highlight two strategies that appeared particularly influential during the 2024 SAGES Annual Meeting.

First, our results suggest that leadership engagement played a meaningful role in framing sustainability as a professional value. The Presidential Address at the SAGES Annual Meeting, which emphasized environmental responsibility, was well received by conference attendees, many of whom expressed feeling inspired and motivated to implement change. Leadership endorsement of this kind may help send a powerful message and encourage broader cultural and operational shifts among the surgical community. This recommendation aligns with findings from a survey study, in which 91% of respondents expressed a desire for greater leadership and guidance from national surgical organizations [18].

**Fig. 1:**
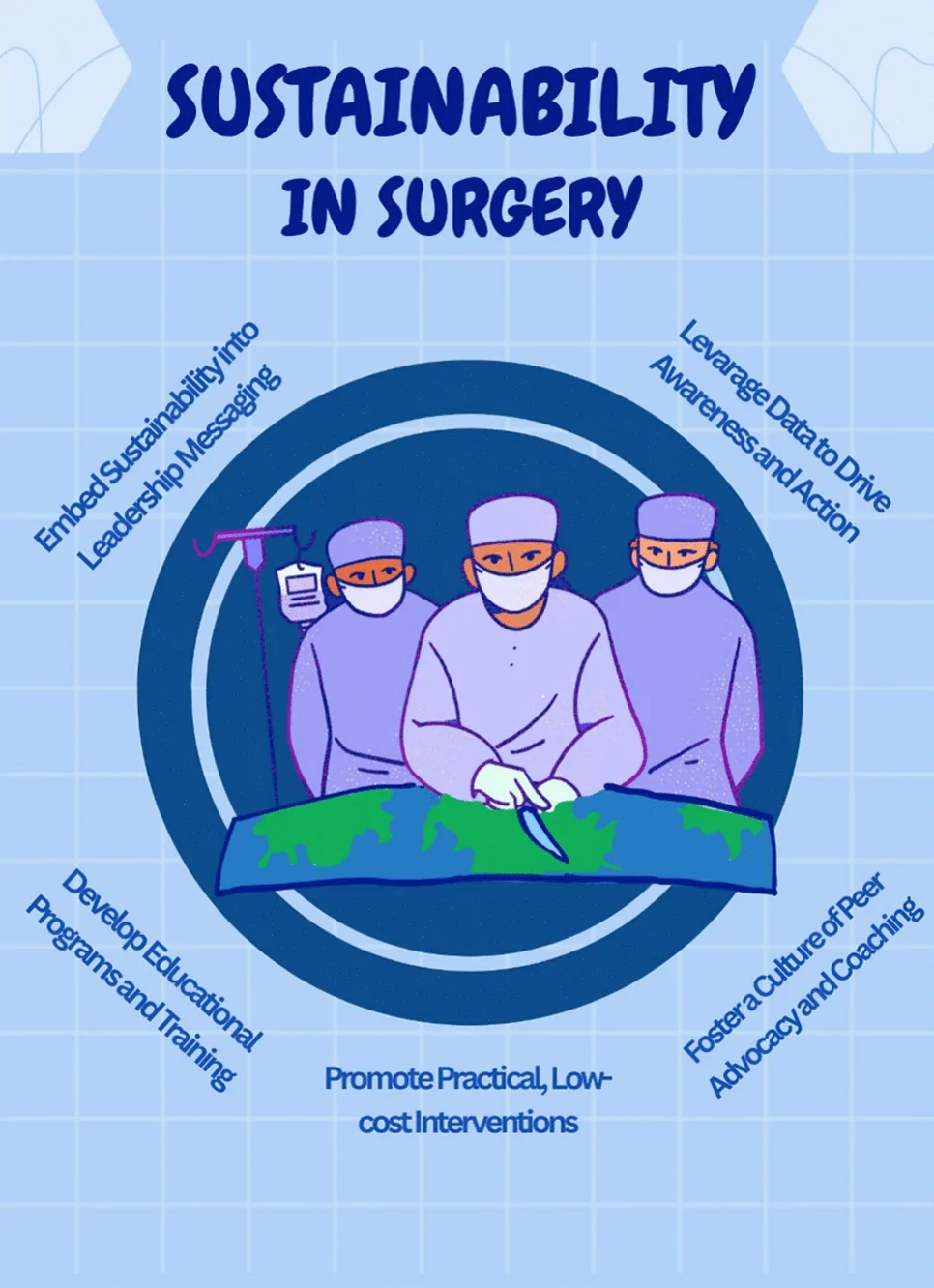
Key messages

Second, the use of data-driven approaches promotes awareness and action. Presenting environmental impact data at the SAGES Annual Meeting was described by participants as an effective way to educate attendees. For some, seeing the data prompted a clear moment of realization that deepened their understanding of the environmental consequences of surgical practice. The inclusion of both quantitative and qualitative data may help foster engagement and encourage thoughtful conversations. This transparency may drive shifts in practice norms, influence decision-making, and support broader culture change. The value of data-driven approaches in advancing environmental sustainability in healthcare has been emphasized by others [20,21]. Our team created a visual representation of the key messages learnt from this study (Fig. 1).

There are several limitations to this study. First, we did not include healthcare professionals other than surgeons and surgical trainees in our sample. Future research should include other perioperative stakeholders, such as OR nurses, administrators, anesthesiologists, and information technology staff, to gain a more comprehensive understanding of sustainability in surgical practice. Second, our interviews were conducted exclusively with attendees of the SAGES conference; thus, the study findings may not necessarily apply to the broader surgical community. Expanding this research to include the surgical community more broadly, as well as ensuring representation from both community and academic settings, would enhance the depth of future research. Participant recruitment via QR code may have introduced selection bias, as individuals who were more technologically inclined or already interested in sustainability may have been more likely to engage. Furthermore, some interviews were conducted via Zoom up to a week after the conference, potentially leading to recall bias regarding the sustainability sessions. Our sample of 18 participants was sufficient, by qualitative standards, to capture the emerging domains and themes [17]. However, the smaller number of residents and fellows compared to attending surgeons limited our ability to explore potential differences in views by training level. Finally, members of SAGES leadership were involved in conceptualization, methodology, resource provision, and supervision of the project. While their leadership roles posed potential for investigator or affiliation bias, they were not involved in conducting interviews or analyzing data, which helped minimize the influence of their organizational positions on the study findings.

## Conclusion

This study reveals a strong commitment among surgeons and surgical trainees to integrate sustainability into surgical practice. Attendees of the sustainability sessions at the 2024 SAGES Annual Meeting emphasized the importance of mindful resource use, data-driven education, and leadership in driving change. Many participants expressed personal motivation to champion sustainable practices within their institutions. These findings illustrate how professional societies can foster awareness and momentum around sustainability in surgery. Future qualitative research may further explore the barriers and facilitators to implementing sustainable practices, offering deeper insight into how such efforts can be effectively supported across diverse surgical settings.

## Supplementary Information

Below is the link to the electronic supplementary material.

Supplementary file1 (DOCX 19 KB)
