# Supplementary material for: Sustainability in surgery: insights from the 2024 SAGES Annual Meeting
Source: Surg Endosc. 2026 Aug 10;40(11):9618–26. doi: 10.1007/s00464-026-13181-x (PMC13754940; doi:10.1007/s00464-026-13181-x)
Supplement: Supplementary file 1 [file 464_2026_13181_MOESM1_ESM.docx]

**Supplemental Material**

**Table A1.** COREQ checklist.

| **Item number** | **Guide questions/description** | **Section** |
| --- | --- | --- |
| **Domain 1: research team and reflexivity** | | |
| **Personal characteristics** | | |
| 1. Interviewer/facilitator | Which author/s conducted the interview or focus group? | Methods, Interview Guide |
| 2. Credentials | What were the researcher’s credentials (e.g. PhD, MD)? | Author List |
| 3. Occupation | What was their occupation at the time of the study? | Methods, Interview Guide |
| 4. Gender | Was the researcher male or female? | Methods, Interview Guide |
| 5. Experience and training | What experience or training did the researcher have? | Methods, Interview Guide |
| **Relationship with participants** | | |
| 6. Relationship established | Was a relationship established prior to study commencement? | Methods, Interview Procedures |
| 7. Participant knowledge of the interviewer | What did the participants know about the researcher (e.g. personal goals, reasons for doing the research)? | N/A |
| 8. Interviewer characteristics | What characteristics were reported about the interviewer/facilitator (e.g. bias, assumptions, reasons and interests in the research topic)? | Methods |
| **Domain 2: study design** | | |
| **Theoretical framework** | | |
| 9. Methodological orientation and Theory | What methodological orientation was stated to underpin the study (e.g. grounded theory, discourse analysis, ethnography, phenomenology, content analysis)? | Methods |
| **Participant selection** | | |
| 10. Sampling | How were participants selected (e.g. purposive, convenience, consecutive, snowball)? | Methods |
| 11. Method of approach | How were participants approached (e.g. face to face, telephone, mail, e-mail)? | Methods, Recruitment |
| 12. Sample size | How many participants were in the study? | Results |
| 13. Non-participation | How many people refused to participate or dropped out? Reasons? | N/A |
| **Setting** | | |
| 14. Setting of data collection | Where was the data collected (e.g. home, clinic, workplace)? | Methods, Interview Procedures |
| 15. Presence of non-participants | Was anyone else present besides the participants and researchers? | N/A |
| 16. Description of sample | What are the important characteristics of the sample (e.g. demographic data, date)? | [Table](https://www.ncbi.nlm.nih.gov/books/n/ukhsdr0834/s2/table/table3/?report=objectonly) 1 |
| **Data collection** | | |
| 17. Interview guide | Were questions, prompts, guides provided by the authors? Was it pilot tested? | Methods, Interview Guide Not pilot tested |
| 18. Repeat interviews | Were repeat interviews carried out? If yes, how many? | No repeat interviews |
| 19. Audio-/visual-recording | Did the research use audio or visual recording to collect the data? | Methods, Interview Procedures |
| 20. Field notes | Were field notes made during and/or after the interview or focus group? | No |
| 21. Duration | What was the duration of the interviews or focus group? | Methods, Interview Procedures |
| 22. Data saturation | Was data saturation discussed? | Yes |
| 23. Transcripts returned | Were transcripts returned to participants for comment and/or correction? | Not relevant in this analysis |
| **Domain 3: analysis and findings** | | |
| **Data analysis** | | |
| 24. Number of data coders | How many data coders coded the data? | Methods, Data Analysis |
| 25. Description of the coding tree | Did authors provide a description of the coding tree? | Methods, Data Analysis |
| 26. Derivation of themes | Were themes identified in advance or derived from the data? | Methods, Data Analysis |
| 27. Software | What software, if applicable, was used to manage the data? | Methods, Data Analysis |
| 28. Participant checking | Did participants provide feedback on the findings? | Not relevant to this method |
| **Reporting** | | |
| 29. Quotations presented | Were participant quotations presented to illustrate the themes/findings? Was each quotation identified (e.g. participant number)? | Yes |
| 30. Data and findings consistent | Was there consistency between the data presented and the findings? | Yes |
| 31. Clarity of major themes | Were major themes clearly presented in the findings? | Yes |
| 32. Clarity of minor themes | Is there a description of diverse cases or discussion of minor themes? | No |
